# Benefit–Cost Analysis of Foot-and-Mouth Disease Vaccination at the Farm-Level in South Vietnam

**DOI:** 10.3389/fvets.2018.00026

**Published:** 2018-02-26

**Authors:** Dinh Bao Truong, Flavie Luce Goutard, Stéphane Bertagnoli, Alexis Delabouglise, Vladimir Grosbois, Marisa Peyre

**Affiliations:** ^1^CIRAD, UMR ASTRE, Montpellier, France; ^2^Faculty of Animal Science and Veterinary Medicine, Nong Lam University, Ho Chi Minh, Vietnam; ^3^Faculty Veterinary Medicine, Kasetsart University, Bangkok, Thailand; ^4^IHAP, Université de Toulouse, INRA, ENVT, Toulouse, France; ^5^Center for Infectious Disease Dynamics, Department of Biology, The Pennsylvania State University, University Park, PA, United States

**Keywords:** animal health economics, benefit-cost analysis, evaluation, financial analysis, foot-and-mouth disease, vaccination

## Abstract

This study aimed to analyze the financial impact of foot-and-mouth disease (FMD) outbreaks in cattle at the farm-level and the benefit–cost ratio (BCR) of biannual vaccination strategy to prevent and eradicate FMD for cattle in South Vietnam. Production data were collected from 49 small-scale dairy farms, 15 large-scale dairy farms, and 249 beef farms of Long An and Tay Ninh province using a questionaire. Financial data of FMD impacts were collected using participatory tools in 37 villages of Long An province. The net present value, i.e., the difference between the benefits (additional revenue and saved costs) and costs (additional costs and revenue foregone), of FMD vaccination in large-scale dairy farms was 2.8 times higher than in small-scale dairy farms and 20 times higher than in beef farms. The BCR of FMD vaccination over 1 year in large-scale dairy farms, small-scale dairy farms, and beef farms were 11.6 [95% confidence interval (95% CI) 6.42–16.45], 9.93 (95% CI 3.45–16.47), and 3.02 (95% CI 0.76–7.19), respectively. The sensitivity analysis showed that varying the vaccination cost had more effect on the BCR of cattle vaccination than varying the market price. This benefit-cost analysis of biannual vaccination strategy showed that investment in FMD prevention can be financially profitable, and therefore sustainable, for dairy farmers. For beef cattle, it is less certain that vaccination is profitable. Additional benefit-cost analysis study of vaccination strategies at the national-level would be required to evaluate and adapt the national strategy to achieve eradication of this disease in Vietnam.

## Introduction

Foot-and-mouth disease (FMD) is recognized to heavily impact livestock production (1). The direct impact of this disease can be classified as two types: visible and invisible losses (1). The visible damages include draft power loss (2), milk production loss (1, 3), abortion (4), death, and decrease in livestock product value (2). The invisible losses include reduction in fertility, delay in the sale of animals and livestock products, change in farm structure (resulting from deaths, decreased parturition rate and delayed sales), and reduced access to the market (1). Moreover, FMD causes additional expenditures (indirect impacts) in disease control such as vaccination, postvaccination monitoring, movements control, diagnostic, and surveillance (1). The impact of FMD is especially meaningful to small producers as it threatens their livelihood and food security (5). In Laos, annual losses due to FMD infection were reported to reach between 16 and 60% of the annual household income (6). In Vietnam, Forman et al. (7) recorded net losses due to FMD ranged between 10 and 32% of the total annual household income. In Cambodia, FMD was shown to reduce the household income by more than 11% every year (2). Vaccination has been recognized as a helpful tool to control FMD and is an essential part of the progressive FMD control pathway from the World Health Organisation (1, 8). In Vietnam, this tool has been applied since 2006 to improve FMD control at a national-level with the objective of reaching eradication by 2020. Currently, the two major FMD serotypes O and A are circulating in Vietnam (9). Vaccines which are currently in use in a biannual strategy are either monovalent (targeting serotype O) or bivalent (targeting serotype O and A). Vaccination is usually implemented twice a year in March–April and September–October (two vaccination campaigns per year). According to the epidemiological situation, provinces of Vietnam are classified into two zones: high-risk (subdivided into control and buffer) and low-risk zones (9, 10). As the risk of emergence is considered to be high in high-risk zone, the program targets mainly those areas. The control zone (high-risk) consists of eight provinces along the northern border, six provinces along the southwest border, between Vietnam and Cambodia, and five provinces located on the border with Laos and the Central Highlands region. The buffer zone (high-risk) consists of 90 provinces adjacent to the control zone. The low-risk zone consists of nine provinces in the Red River Delta region, four important export provinces along the North Central Coast (Nghe An, Thanh Hoa) in the Red River Delta region (Ninh Binh, Vinh Phuc), nine provinces in the Mekong Delta region, and three provinces in the South-East region and Ho Chi Minh City (9, 10). Vaccination is partly supported by the government. In the control and the buffer zones, vaccine fees are financed up to 100% (free vaccine twice a year) and 50% (free vaccine for the first campaign in March–April, vaccine bought by farmers for the second campaign in September–October) of their costs, respectively, by the national budget, while the labor cost of the commune’s veterinarian is paid for by the local authorities. In low-risk zones, these fees are paid for by the local authorities (9, 10). However, this strategy is facing many logistical and economic constraints, i.e., lack of strict implementation and sustainability at the farm-level and reduced perception of FMD risk after several years without outbreak. Therefore, its effectiveness, in terms of vaccine coverage and disease control, has not been achieved, i.e., outbreaks are still continuously recorded (9, 10).

Benefit–cost analysis (BCA) is a commonly used analytical framework that supports the decision-making process in animal disease control (11). When the farmers face a particular livestock health issue, BCA allows comparisons between the cost incurred and the benefit derived from the different available control methods in terms of financial return (11) or livelihood and overall wellbeing (12). The outputs of a BCA would not only foster the vaccination policy review and modification at a national-level but also provide evidence which can encourage farmers’ participation in the campaign. In Ethiopia, it was reported that the national targeted vaccination program was the most economically beneficial strategy, with a median benefit–cost ratio (BCR) of 4.29 (13). In Cambodia, Young et al. (14) estimated that the implementation of a biannual FMD vaccination campaign in large ruminants during 5 years had a BCR of 1.4 (95% CI 0.96–2.20). In South Sudan, the BCR of FMD vaccination was estimated at 11.5 (3). Despite its relevance, no BCA for FMD vaccination at the farm-level has so far been completed in Vietnam. The aim of this study was to analyze the FMD financial impact at the farm-level in Vietnam and the BCR of the vaccination program to address this knowledge gap and better inform policy decision.

## Materials and Methods

### Study Area

**Figure 1:**
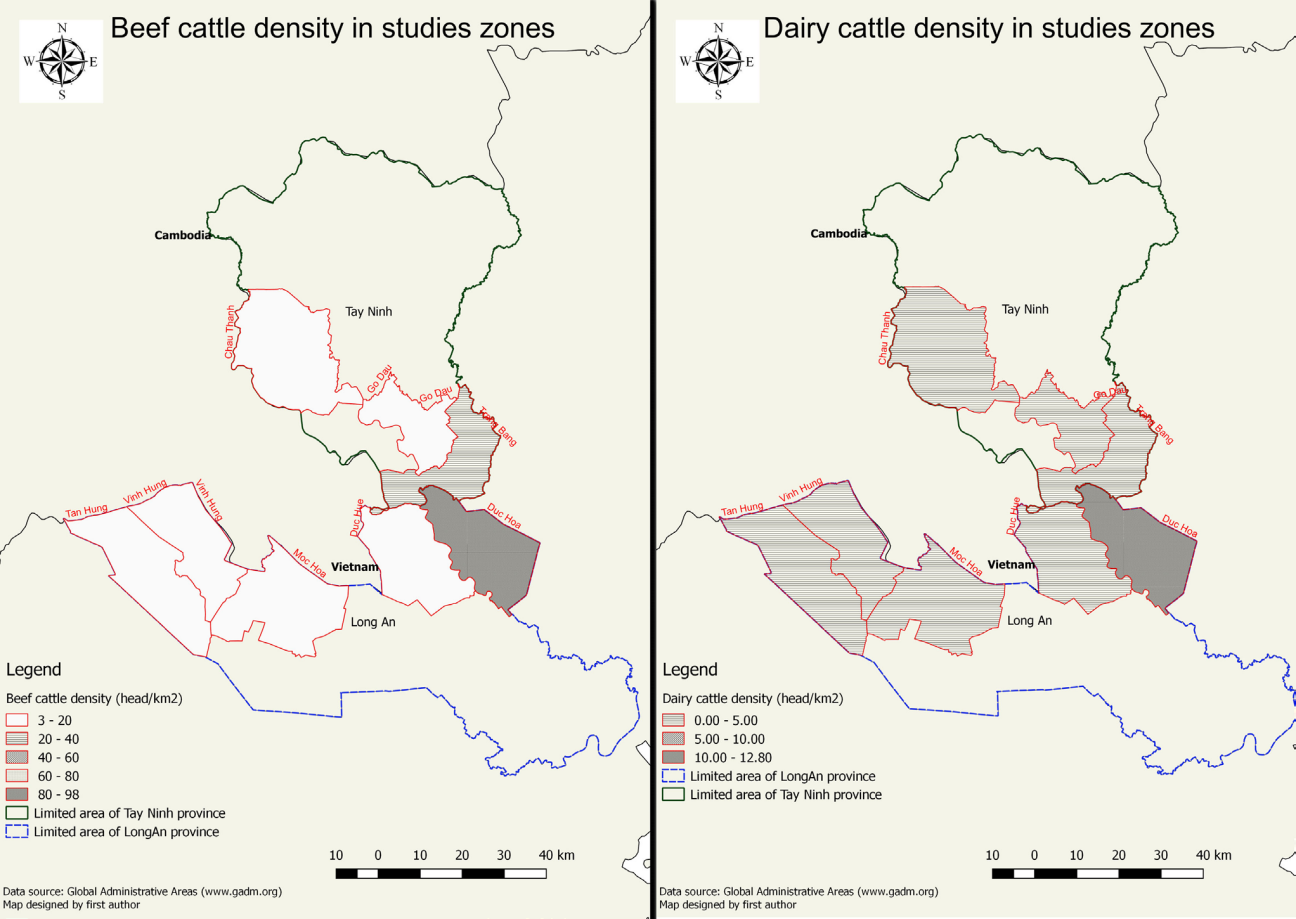
Cattle population density in eights districts under study (left: beef cattle; right: dairy cattle).

The study was performed in five districts of Long An province (i.e., Tan Hung, Vinh Hung, Kien Tuong, Duc Hoa, Duc Hue) and three districts of Tay Ninh province (i.e., Trang Bang, Chau Thanh, Go Dau). These districts were selected, in agreement with the sub-Department of Animal Health of the provinces under study, based on the importance of their livestock production, their proximity to the Cambodian border, the importance of animal movements from these districts to other provinces and countries, and their location in the high-risk area for FMD. The limited area of study as well as cattle population density were visualized in Figure 1. Two types of survey were implemented in the field. The general survey aimed to collect farm production and farm management and was conducted in eight districts of two provinces as mentioned above. The second survey named financial impact survey was done in only two districts of Long An province in the framework of another study implemented in the same period ((15), submitted manuscripts).

### Data Collection Process

A questionnaire-based survey (general survey) was performed to collect general information on farm production and farm management practices, such as the number of cattle per farm, the number of calves and adult cattle per farm, the unit price of one dose of a bivalent vaccine, the cattle live weight price per kg, the price of an insemination service, and the milk price per liter. This survey was performed from June to October 2014 in the eight districts of the study area as mentioned above, with the help of a group of 15 veterinary students from Nong Lam University, Ho Chi Minh City. The students were trained about the questionnaire structure and face-to-face interview method by two certified lecturers from Nong Lam University 1 month before performing the field survey. Farmer within two types of cattle production (dairy and beef) in eight districts mentioned above were invited to participated in questionnaire-based survey. The total number of interviewed farms per district was based on the cattle population density in each district. A stratified sampling for farms selection was performed based on the type of cattle production (dairy or beef) with a limit of 10 questionnaires per production type per village.

Data about the financial impact of FMD (relative costs of control FMD case in cattle at the farm-level) were collected in farms with FMD suspicion which were detected during study period (animal having clinical signs of the disease that were recognized by farmer). Indeed, a series of focus group and individual interviews took place from November 2015 to April 2016 in the framework of a study on the topic of participatory surveillance in sentinel villages in Duc Hoa and Duc Hue district of Long An province. Focus group interviews of 10–15 farmers per village were implemented to identify farms present suspected cases of FMD. Those suspected farms were then being the subject to individual semi-structured interviews to collect data on FMD financial impact. The results of the participatory surveillance study were reported apart, and financial impact data collected from infected farms were presented and used in this paper. In those farms, general data on disease management, control methods, disease impact, and all related costs were first collected using a standardized questionnaire. The first part of the questionnaire included general questions on the number of cattle at risk, number of disease cases, number of deaths due to the disease, number of premature slaughters, number of cattle destroyed, number of cattle vaccinated, vaccine type used, and actual vaccination practices applied in the farm. The second part of the survey contained questions on the financial costs associated with FMD infections. Farmers were asked to describe the cost associated with each control measure applied in their farm such as treatment with modern and/or local medicine, disinfection, emergency sale or slaughter of infected (dead) animals, emergency vaccination of unvaccinated cattle in case of outbreak, as well as the financial cost of disease-related increase in abortion and decrease in milk production. The value of an infected (dead) animal was based on the price paid to farmers by traders at the time of the survey. The value of new-born calves was estimated by farmers based on feed intake and the sale price of healthy calves sold at 3 months of age.

### Calculation of Incidence Rates and Incidence Risks of FMD in Cattle Farms in the Study Area

It was assumed that cattle infected once by FMD did not get infected later in their productive life. A FMD sero-prevalence of 60% at the animal-level in infected herds was measured in the study area (unpublished data). It was assumed that antibodies against FMD are detected in cattle up to 3 years postinfection (16).

The incidence rate of FMD was calculated using the following formula:

(1) $$\lambda =\frac{-\text{log}(\text{1}-{\text{p}}_{\text{x}})}{\text{x}}.$$

With: λ being the herd incidence rate of FMD, p_x_ the measured sero-prevalence in the cattle population, x the duration of FMD immunity in cattle (the period during which FMD antibody are detected after infection).

The proportion of slaughtered cattle that have been infected during their whole lifetime is:

(2) $${\text{p}}_{\text{T}}=1-{\text{e}}^{-\text{λT}}.$$

With: T being the average duration of a cattle productive life (or age at slaughter) (6 years in dairy cattle, 12 years in beef cattle).

The fraction of the cattle population of a given cattle farm being infected by FMD over 1 year (the number of cattle infected over 1 year divided by the total herd size) is:

(3) $${\text{p}}_{\text{y}}=\frac{1-{\text{e}}^{-\text{λT}}}{\text{T}}.$$

The proportion of calves being infected by FMD over 1 year in a given farm (the number of calves infected over 1 year divided by the total calve population of the herd) is:

(4) $${\text{p}}_{\text{y}}=\frac{1-{\text{e}}^{-\lambda {\text{T}}_{\text{c}}}}{{\text{T}}_{\text{c}}}.$$

With T_c_ the age cattle become adults (the age of first calving for females).

The proportion of adult cattle being infected by FMD over 1 year in a given farm (the number of adult cattle infected over 1 year divided by the total adult cattle population of the herd) is:

(5) $${\text{p}}_{\text{ya}}=\frac{{\text{e}}^{-\lambda {\text{T}}_{\text{c}}}-{\text{e}}^{-\lambda {\text{T}}_{\text{c}}}}{\text{T}-{\text{T}}_{\text{c}}}.$$

### Partial Budget Analysis at Farm-Level

The analysis was based on the methodological framework proposed by Dijkhuizen et al. (17) and Rushton et al. (18), modified and adapted to the study context. The components used in the partial budget analysis are described below. The analysis includes additional revenue, foregone revenue, additional costs, and saved costs, compares “*status quo*” scenario with no FMD vaccination to an alternative scenario where FMD vaccination is applied twice a year. The formula for calculation of additional costs, saved costs, additional revenue, and foregone revenue as well as their subcomponents and used variables are detailed in Table 1.

**Table 1:** Formula and variables used in the partial budget analysis of foot-and-mouth disease (FMD) vaccination in South Vietnam.

Formula and variables
Additional costs = labour + vac = (labour.ani + p.vac) × N.j.k × n.p

Labour labour cost of vaccination; vac: expenditure in vaccine purchase; labour.ani: labour cost per injection per cattle; p.vac: unit price of one dose of a bivalent vaccine; N.j.k: number of cattle per farm depending on scale *j* and farm type *k*; n.p: number of injections per year

$\begin{array}{cc} \text{Saved costs}= & \text{Treat.cost.k}+\text{rep.a.d}+\text{rep.c.d}+\text{e.vac.c}+\text{Ser.loss}\\ & +\text{Treat.cost.k}={\text{p}}_{\text{y}}\times (\text{Treat.mod.k}+\text{Treat.loc.k})\times \text{N.j.k}\times \text{morb.k}\\ & +\text{rep.a.d}={\text{p}}_{\text{ya}}\times (\text{p.cow.h}-\text{p.cow.d})\times \text{N.a.jk}\times \text{Mort.k}\\ & +\text{rep.c.d}={\text{p}}_{\text{yc}}\times (\text{p.calf.h}-\text{p.calf.d})\times \text{N.ca.jk}\times \text{Mort.k}\\ & +\text{e.vac.c}={\text{p}}_{\text{ya}}\times (\text{labour.ani}+\text{p.vac})\times (\text{N.jk}-\text{N.ca.jk})\times 2\times \text{Morb.k}\\ & +\text{Ser.loss}={\text{p}}_{\text{ya}}\times \text{N.a.jk}\times \text{per.cow.ges}\times \text{Abor.FMD}\times \text{no.ser.ges.i}\\ & \times \text{P.ser}\times \text{Morb.k} \end{array}$

2: vaccine injections are performed at 28 days interval; e.vac.c: cost of emergency vaccination over the considered period; Morb.k: morbidity rate in case of FMD outbreak; N.a.jk: number of adult cattle per batch; N.ca.jk: number of calf per batch; N.j.k: the number of animal per batch (all cattle in the same production cycle); (N.jk − N.ca.jk): number of adult animal in scale *j* and farm type *k* in emergency vaccination; no.ser.ges.i: the average number of artificial or natural insemination service performed by veterinarians for each cow to become pregnant; p.cow.h: value of a healthy adult cattle p.cow.d: value of a dead or treated cattle p_yc_: proportion of calves being infected by FMD over 1 year (calculated using Eq. 4), p.calf.h: value of a healthy calf, p.calf.d: value of a dead/treated calf; p_y_: proportion of a given cattle farm being infected by FMD over 1 year (calculated using Eq. 3), p_ya_: proportion of adult cattle being infected by FMD over 1 year (calculated using Eq. 5); P.Ser: average price of an insemination service. rep.a.d(rep.c.d): the cost of replacing adult cattle (calf) in case of death over the considered period; Ser.loss: the cost of additional insemination services used due to FMD over the considered period; Treat.cost.k: cost of FMD treatment with modern and indigenous medicine over the considered period; Treat.mod.k (Treat.loc.k): cost of treatment with modern (indigenous) medicine per affected cattle during the outbreak period

$\begin{array}{cc} \text{Additional revenue}= & \text{M.prod}+\text{W.h.a}+\text{W.extra}+\text{Abor.red}\\ & +\text{M.prod}={\text{p}}_{\text{ya}}\times \text{t.ill}\times \text{M}\times \text{P.milk}\times \text{N.a.jk}\times \text{per.cow}\times \text{Morb.k}\\ & +\text{W.h.a}={\text{p}}_{\text{y}}\times \text{t.ill}\times \text{dwg}\times \text{p.liveW}\times \text{N.jk}\times \text{Morb.k.}\\ & +\text{W.loss}={\text{p}}_{\text{T}}\times \text{cull.rate}\times \text{per.W.loss}\times \text{W.cow.h}\times \text{p.liveW}\\ & \;\times \text{N.jk}\times (\text{Morb.k}-\text{Mort.k})\\ & +\text{Abor.loss}={\text{p}}_{\text{ya}}\times \text{N.a.jk}\times \text{per.cow.ges}\times \text{no.calves.prod}\\ & \;\times \text{Abor.FMD}\times \text{p.n.calf}\times \text{Morb.k} \end{array}$

Abor.FMD: the increase in abortion rate due to FMD infection, Abor.red: additional cattle raised value due to less abortion cull.rate being the proportion of the cattle farm being culled each year (it is the inverse of the age at maturity—$\mathrm{cull}\;\text{rate}=\frac{1}{T}$); dwg: average daily weight gain; M: average quantity of milk produced per lactating cow per day; M.prod: additional milk production value; $\text{no.calves.prod}=\frac{\text{duration of a year in day}}{\text{overall mean of calving interval in day}\;(\text{ci})}$: Number of calves produced per cow in 1 year;

N.a.jk: number of adult cows in farm; P.milk: price of one liter of milk; per.cow.lac: percentage of lactating cows in the farm (including cow with pregnant and lactating at the same time); p.liveW: price of a live weight in kilogram; p_T_: proportion of slaughtered cattle having been infected during their whole lifetime (calculated in Eq. 1); per.W.loss:average percentage of weight loss of cattle due to FMD; p.liveW: live weight price (per kilogram); per.cow.ges: percentage of adult cattle which are gestating cow in the farm; p.n.calf: price of a new-born calf estimated by farmer; t.ill: average duration of illness due to FMD; W.h.a: additional weight gain value; W. extra: additional cattle raised value due to lower mortality; W.cow.h: average weight of a healthy cow at sale time in kilogram.

$\begin{array}{cc} \text{Foregone revenue}= & \text{inc.a.d}+\text{inc.c.d}\\ & +\text{inc.a.d}={\text{p}}_{\text{ya}}\times \text{p.cow.d}\times \text{N.a.jk}\times \text{Mort.a}\\ & +\text{inc.c.d}={\text{p}}_{\text{yc}}\times \text{p.calf.d}\times \text{N.ca.jk}\times \text{Mort.c.} \end{array}$
inc.a.d: income of selling dead/sick adult cattle; inc.c.d: income of selling dead/sick calves.

Additional costs represent costs incurred in the alternative scenario that are not present in the “*status quo*” scenario. It includes vaccine price (vac) and labor cost of vaccination practice (labour) that farmer needs to pay. Extra feed and labor cost of farming more cattle in farm because of the reduced mortality and drop in abortion was not included in our analysis as all animals were assumed to be replaced in “*status quo*” scenario.

Saved (avoided) costs represent costs incurred in the “*status quo*” scenario that are avoided in the alternative scenario. It includes cost of disease treatment (Treat.cost.k) with modern and local medicine per cattle, cost of replacing adult cattle (rep.a.d) and calves (rep.c.d) in case of death over the considered period, cost of emergency vaccination (e.vac.c), and cost of additional insemination services (ser.loss). Cost of movement restriction was excluded because feed intake during delay time could not be collected. Cost of disinfection was also excluded because the relative data could not be quantified.

Additional revenue represents the revenue generated in the alternative scenario which is not present in the “*status quo*” scenario. It includes revenue gain from additional milk production from healthy cattle (M.prod) from selling healthy cattle at higher price due to higher weight compared to lower weight of infected (weight lost during sick period) (W.h.a), additional cattle raised and sold when there is less mortality (W.extra), and less abortion (Abor.red) due to FMD infection. We did not include the additional revenue from additional milk production resulting from the reduction of cows’ mortality. Indeed, we did not have the necessary data on the additional quantity of feed consumed to sustain this increased milk production.

Subsidies of government, which generally covered between 50 and 100% of the vaccination costs, were not taken into account in the calculation since the analysis was done at farm-level, without considering any contribution from the government, which returned a more conservative result.

Foregone revenue represents the revenue generated in the “*status quo*” scenario which is not present in the alternative scenario. It includes revenue lost due to adverse impacts of vaccination on productivity such as decreased milk production, decreased daily weight gain, and impact on reproduction such as abortion due to stress caused by bad practice. It also includes the revenue from selling dead or sick cattle and calves (inc.a.d + inc.c.d) at lower price. As data were missing foregone revenue due to adverse vaccination effects vaccination was considered to be null. It was also assumed the vaccination was perfectly implemented, and did not cause any adverse effect due to stress.

### Benefit–Cost Analysis

Partial budget analysis was used to estimate the benefits (additional revenue and saved costs) and costs (additional costs and revenue foregone) of using vaccination method of one given farm to prevent FMD over a 1-year period. The total benefit of the vaccination program is the sum of the additional revenue and saved costs while the total cost is the sum of the foregone revenue and additional costs.

The net present value (NPV) of the proposed change in disease control strategy observed in alternative scenario compared to “*status quo*” scenario was calculated on an individual farm for the period of 1 year as follow:

(6) $$\begin{array}{c} \text{Net present value}=(\text{saved cost}+\text{additional revenue})\\ \;-(\text{additional cost}+\text{foregone revenue}). \end{array}$$

The BCR between alternative scenario and “*status quo*” scenario was also computed on an individual farm using following formula:

(7) $$\begin{array}{c} \text{Benefit}-\text{cost ratio}=(\text{saved cost}+\text{additional revenue})\\ \;\text{/}(\text{additional cost}+\text{foregone revenue}). \end{array}$$

Benefit–cost ratio was calculated for three types of production: large-scale and small-scale dairy farm and small-scale beef farm. The distinction in scale was based on the classification used in national program of vaccination. In this program, farm present less than twenty animals was classified as small, farm within more than twenty animal was considered as large (10).

### Sensitivity Analysis

The sensitivity analysis for benefit–cost of FMD vaccination was performed by changing vaccination cost and market prices of sold cattle and milk. This analysis was performed to understand the variation in benefit–cost and the influence of the variance of these parameters on the BCR associated with FMD vaccination. Eight scenarios (C1–C8) were tested by changing vaccination cost and/or market value of milk and slaughtered cattle (Table 2). In C1 and C2, vaccination cost was increased by 25 and 50%, respectively. In C3 and C4, the market price of cattle and milk were decreased by 10 and 20%, respectively. From C5 to C8, changes in both parameters were performed. The increase in vaccination cost of 25% and 50% was based on hypothesis that farmer would rather use trivalent vaccine in the future if the presence of the third serotype would be confirmed (vaccination cost increase of 25%) or farmer would practice vaccination more than twice a year (vaccination cost increase of 50%). The decrease in market value of 10 and 20% was based on market tendency of milk and meat product. The milk price tends to be decreased because of excess supply source and meat price also decreased because of the competition of imported meat from India and Australia.

**Table 2:** Proposed scenarios for sensitivity analysis of benefit–cost ratio.

Scenario	Vaccination Cost	Milk and cattle market value
C1	Increased by 25%	NA
C2	Increased by 50%	NA
C3	NA	Decreased by 10%
C4	NA	Decreased by 20%
C5	Increased by 25%	Decreased by 10%
C6	Increased by 25%	Decreased by 20%
C7	Increased by 50%	Decreased by 10%
C8	Increased by 50%	Decreased by 20%

*NA, not applicable*.

### Assumptions Used in the Cost–Benefit Analysis

Some parameters used in the BCA were taken from the literature (Table 3) because those parameters could not be collected from the field studies. It was assumed that all dairy and beef farms used Holstein-Friesian crossbreeds and Red Sindhi crossbreeds, respectively, based on Vo (19) and Hoang (20). The duration of the productive life of dairy and beef cattle were considered to be 6 and 12 years, respectively. Subsequently, the BCA was calculated for 1 year but took into consideration the duration of the productive life of dairy and beef cattle in the calculation of FMD incidence risks to be able to compare the result for the two types of production. Milk price was based on its quality and was considered as being the same for every lactating cow. Vaccination was considered to be applied within the best practices and to be match with OIE standard for FMD vaccination. Vaccine should contain at least three PD50 (50% of protective Dose) which corresponded to 78% protection using protection against generalization test (21). Vaccination was considered not causing stress in cattle and, therefore, not impacting abortion rate. Only acute FMD was taken into consideration in this analysis while chronic FMD was excluded. The average FMD mortality in adult cattle was estimated at 7.3% (2) instead of the observed value in the field (12%) after consulting expert’s opinion. These values were also added in sensitivity analysis due to uncertainty nature of the data.

**Table 3:** Input data and references used to estimate foot-and-mouth disease (FMD) vaccination benefits and costs for farmers.

Input data (unit)	Production type		Description and/or data sources	Abbreviation
	Dairy cattle farms	Beef cattle farms		
Abortion rate due to FMD (%)	10 ± 2.3^a^		Senturk and Yalcin (4)	Abor.FMD
Average number of milk produced per cow per day (liter)	11.4 ± 0.3^a^	NA	Le and Nguyen (22)	M
Average weight of a healthy animal (kg)	418 ± 6.25^a^	284.6 ± 35^a^	Based on Dinh (23), for beef, Le and Nguyen (22) for dairy	W.cow.h
Average weight loss when infected (%)	24 ± 1.16^a^		Young (2)	per.W.loss
Duration of illness (days)	11.1 ± 1.33^a^		Young (2)	t.ill
Estimated mean daily weigh gain (kg/day)	0.5^c^	0.36^c^	Dinh (24) for dairy, Dinh (23) for beef	Dwg
Median calving interval (days)	441^c^	390^c^	Dinh (24) for dairy, Dinh (23) for beef	Ci
Age of first calving (years)	2.19^c^	2.13^c^	Dinh (24) for dairy, Dinh (23) for beef	T_C_
Number of average service for a cow being gestation (time)	2 ± 0.11^a^		(22)	no.ser.ges.i
Percentage of lactation cow in farm (%)	50^c^	NA	Vo et al. (25)	per.cow.lac
Percentage of pregnant cow in farm (%)	58^c^	56.31^c^	Calculation based on data of Vo et al. (25) for dairy, Dinh (23) for beef	per.cow.ges
Mortality rate in a farm (%) adult cattle^f^	7.3^c,d^–12^c,e^		(2)	Mort.a
Incidence rate of FMD at farm level	30 (26.2–33.7)^b^	30 (26.2–33.7)^b^	(26)	λ
Duration of FMD immunity in cattle (year)	3^c^	3^c^	(16)	*x*
Average duration of a cattle productive life (or age at slaughter)	6^c^	12^c^	Author estimation	T

*NA, not applicable*.

*Type of probability distribution*.

*^a^Normal distribution: mean ± SD*.

*^b^Normal distribution: mean (CI 95%)*.

*^c^Data available as mean value only*.

*^d^Value from literature*.

*^e^Value issued from financial impact survey*.

*^f^Two values were used in sensitivity analysis*.

### Data Analysis

All analysis was performed using R software version 3.3.1. A framework of calculation NPV and BCR which included functions and formula described above and in Table 1 was developed in R environment for three production types. The uncertainty over the value of the parameters used in the analysis was addressed through a Monte Carlo procedure. The probability distribution of the NPVs and BCRs where obtained by sampling 1,000 values of parameters from their respective assumed probability distributions, using a random Latin Hypercube sampling procedure (Carnell R. lhs: Latin Hypercube Samples. R package version 0.14 2016. Available from: https://CRAN.R-project.org/package=lhs.). According to the information available, different types of data were used in the analysis. Triangle distribution data was available for value of a healthy calf/cow, value of a dead or after treatment calf/cow, vaccination labor cost, cost of treatment with indigenous/modern medicine, number of calves/adults cattle per farm according to each production types, number of animal per farm, mortality rate in a farm for calf. Normal distribution is seen in data of abortion rate due to FMD, volume of milk produced per cow per day, weight of a healthy animal, weight loss when being infected, duration of illness, number of service for a cow being gestation, incidence rate of FMD at the herd-level. Value of a new-born calf and mortality rate of adult animal were available only in uniform distribution (more details could be found in Tables 3–6). BCRs were consistently higher than 1 indicated that the considered investment in FMD vaccination was worthwhile. Data were calculated using “reshape2” (27) and reported using “knitr” package (28).

**Table 4:** Description of the animal production parameters from the study area extracted from the general survey.

Variables	Dairy cattle farm	Beef cattle farm	Abbreviation
Number of adult cattle per farm, small-scale	8 (1–19)^a^	2 (1–16)^a^	N.a.jk
Number of adult cattle per farm, large-scale	19 (13–41)^a^	NA	
Number of calf per farm, small-scale	1 (1–8)^a^	11 (1–10)^a^	N.calf. jk
Number of calf per farm, large-scale	1 (1–9)^a^	NA	
Number of animal per farm, small-scale (<20 heads)	12 (2–20)^a^	2 (1–16)^a^	N.j.k
Number of animal per farm, large-scale (>20 heads)	25 (21–50)^a^	NA	

*NA, not applicable; type of probability distribution: ^a^triangle distribution: mode (min–max)*.

**Table 5:** Description of the estimated parameters used for the benefit-cost calculation of foot and mouth disease extracted from the general survey.

Parameters	Dairy cattle farms	Beef cattle farms	Abbreviation
Proportion of slaughtered cattle having been infected during their whole lifetime	0.84	0.97	p_T_

Proportion of a given cattle farm being infected by foot-and-mouth disease (FMD) over 1 year	0.14	0.08	p_y_

Proportion of calves being infected by FMD over 1 year	0.22	0.22	p_yc_

Proportion of adult cattle being infected by FMD over 1 year	0.09	0.05	p_ya_

**Table 6:** Description of the parameters used for the benefit-cost calculation of foot-and-mouth disease extracted from the financial impact survey.

Input data	*n*	Dairy cattle farm	Beef cattle farm	Abbreviation
Cost of treatment with indigenous medicine per animal (kVND/head)	46	100 (5–875)^a^		Treat.loc.k
Cost of treatment with modern medicine per animal	90	300 (30–2,300)^a^		Treat.mod.k
Value of a dead calf or after treatment (kVND/head) ≤6 months	13	0 (0–14,800)^a^		p.calf.d
Value of a dead or sold cow after treatment (kVND/head)	15	45,000 (700–45,000)^a^		p.cow.d
Value of a healthy calf (kVND/head) ≤6 months	11	10,000 (10,000–19,000)^a^		p.calf.h
Value of a healthy cow (kVND/head)	15	35,000 (18,000–55,000)^a^		p.cow.h
Labor cost per injection (kVND/head)	NA	4 (4–30)^a^		labor.vac
Morbidity in a farm (%) (*n* = 129)	129	79	54	Morb.k
Mortality rate in a farm (%) for calf	8	18 (0–50)^a^		Mort.c
Number of possible calves produced per cow in 1 year	NA	0.83	0.94	no.calves.prod
Price of 1 dose of bivalence vaccine (kVND/dose)	NA	37		p.vac
Price of 1 kg live weight (kVND), value in Dec 2015	NA	140		p.liveW
Price of one service (kVND/time)	184	173		P.Ser
Price of 1 liter of milk (kVND/liter), value in Dec 2015	NA	13.5	NA	P.Milk

*Type of probability distribution: ^a^triangle distribution: mode (min–max)*.

*kVND, thousands of Vietnam Dong (Vietnamese currency)*.

### Ethical Considerations

Our study was approved by the local authorities (sub-Department of Animal Health of Long An). Ethical considerations were properly taken into account. In Vietnam, this study was considered as a common study on animal health and therefore no ethical committee is provided by the national authorities.

Informed consent was obtained from all farmers included in the study. As for each individual interview, each participant signed a written consent to be part of this study.

## Results

### Partial Analysis of FMD Vaccination

#### General Survey

Livestock production data which were collected by questionnaire from 49 small-scale dairy farms, 15 large-scale dairy farms, and 249 beef farms located in 37 villages of eight districts were summarized in Table 4. While beef farms were widely distributed in eight districts under study, dairy farms were mainly practiced in Duc Hoa district of Long An province and Trang Bang district of Tay Ninh province.

#### Financial Impact Survey

A total of 69 focus group interviews were organized in 32 villages of Duc Hoa and Duc Hue districts with the participation of 702 farmers. 129 farms located in 14 villages were then detected as suspected farms and being subject for individual interview using financial impact survey. The investigation demonstrated that in case of being infected by FMD, 43.8% of the cattle in the three production types received treatment with only modern medicine rather than local medicine (11.5%) or with both modern and local medicine (20.9%). Local medicine was especially used in the beef production type (92.6% of cases). The incidence rates and incidence risks of FMD in cattle farm estimated from the collected data and literature using Eqs 1–5 were presented in Table 5. Other data on the financial impact of FMD outbreaks at the farm-level was summarized in Table 6.

#### Partial Analysis of FMD Vaccination

The NPV of FMD vaccination versus “*status quo*” scenario was always positive in dairy farms. However, in beef farms, the 95% CI of the NPV encompassed 0, meaning that the NPV of vaccination could be negative (Table 7), or it is not sure whether vaccination is profitable in beef farms. The mean NPV was highest for the large-scale dairy farms [44,438 kVND per year (95% CI 25,175–65,467)], followed by small-scale dairy farms [15,664 kVND per year (95% CI 4,703–27,202)], and beef farms [1,499 kVND per year (95% CI −2,896 to 5,142)] (Table 7). The average value of additional revenue in large-scale dairy farms was 48,548 kVND per farm per year, which was 2.8 times higher than in small-scale dairy farms and around 19 times higher than in small-scale beef farms.

**Table 7:** Partial budget analysis results according to the different production types (small-scale dairy cattle farms, large-scale dairy cattle farms, and small-scale beef cattle farms).^a^

	Small-scale dairy farms	Large-scale dairy cattle farms	Small-scale beef cattle farms
Additional cost (kVND)	1,120 (459–1,922)	3,193 (2,075–5,289)	691 (177–1,548)
Foregone revenue (kVND)	3,195 (868–6,401)	7,383 (1,542–14,437)	1,731 (238–3,966)
Saved cost (kVND)	2,739 (−17 to 6,227)	6,466 (−352 to 14,633)	1,346 (−814 to 3,667)
Additional revenue (kVND)	17,240 (6,523–26,603)	48,548 (33,407–69,647)	2,576 (580–5,609)
Net present value (kVND)	15,664 (4,703–27,202)	44,438 (25,175–65,467)	1,499 (−2,896 to 5,142)

*^a^Result of Monte Carlo Simulation: mean (CI 95%)*.

*kVND: thousands of Vietnam Dong (Vietnamese currency)*.

### BCA of FMD Vaccination and Sensitivity Analysis

**Figure 2:**
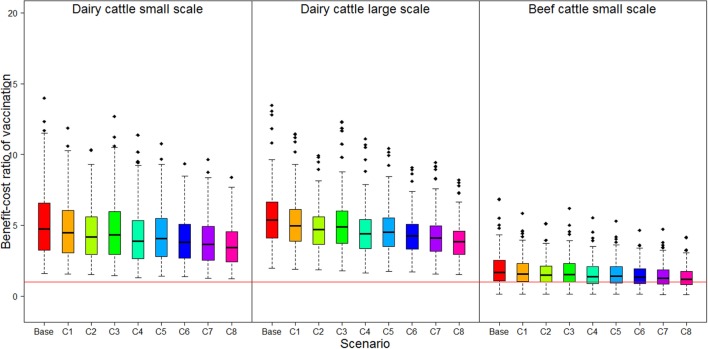
Benefit–cost ratio and sensibility analysis results of vaccination strategy for foot-and-mouth disease in three production types. Base: benefit–cost ratio (BCR) in real situation, C1–C8: proposed scenarios for sensitivity analysis detailed in Table 2. Red horizontal line: threshold of BCR.

All the parameters estimated and used in the analysis are presented in Table 7. The BCR was highest in large-scale dairy farm (5.74 95% CI 2.83–12.34), followed by small-scale dairy farm [5.24 (95% CI 1.88–11.61)], and it was lowest in small-scale beef farm [1.95 (95% CI 0.31–4.91)] (Figure 2; Table S1 in Supplementary Material). The sensitivity analysis showed that the effect of varying the vaccine cost on the resulting vaccination BCR was higher in beef farms than in dairy farms. However, the effect of varying market prices on the resulting vaccination BCR was higher in dairy farms than in beef farms (Figure 2; Table 1 in Supplementary Material). For three production types, changes in market value had more impact on the BCR than changes in vaccination cost. The BCR of vaccination in dairy farms was always higher than 1 in the 8 proposed scenarios—increased vaccination costs and/or decreased milk and/or cattle price. This implies that even at high vaccine price and low market value, FMD vaccination was still profitable. In small-scale beef farms, however, the 95% CI of the BCR included 1 in the 8 proposed scenarios, meaning that the FMD vaccination could be profitable or not depending on the value of the parameters.

## Discussion

In Vietnam, an important fraction of the national budget for FMD prevention and control is dedicated to vaccination, including delivery costs and subsidies for vaccine purchase, which vary from 50 to 100% of the vaccine price in high-risk areas. However, outbreaks are still continuously recorded (9). This observation raises concerns over the effectiveness of the vaccination program and its acceptability at the farm-level. The BCA demonstrated the financial interest for dairy cattle farmers of using vaccination to control FMD as, regardless of the used scenario, FMD vaccination was always profitable. For beef farmers, however, the financial profit derived from vaccination appeared weaker and uncertain, as the BCR could be higher or lower than 1 depending on parameters’ value (e.g., the cost of replacing adult cattle or calf in case of death—rep.a.d or rep.c.d). The output of this study might be used to motivate dairy farmers to participate in vaccination campaigns. It also suggests that high FMD vaccination coverage may be more difficult to reach in the beef cattle sector than in the dairy cattle sector since the expected financial profit from FMD vaccination is much lower in farms of the former category. Yet, sufficient vaccination coverage needs to be reached in both sectors in order to control the disease at the national-level. The latter information may be used by decision makers to refine the national program of prevention and control of FMD in Vietnam. One way of improvement would be, for example, to provide stronger support to FMD vaccination in beef farms (e.g., with subsidies).

Decision to vaccinate depends on other factors such as real and perceived effectiveness of vaccination (11). Perception of farmers may vary with time and maintaining farmers’ motivation to vaccinate is challenging since farmers always balance the risk of adverse consequences of diseases and cost of prevention. During the 6–12 years of cattle life, farmers can stop using vaccination at any moment if they perceive the probability of infection to be low enough; based on the information they get through official reports, media, and other sources of information. FMD surveillance data showed that in Vietnam, peaks of FMD outbreaks occurred every 2–3 years, and were negatively correlated with FMD vaccination coverage (26). During the survey we conducted, some farmers reported they refused to use vaccines because of their potential adverse effect on cattle such as increased risk of abortion, growth delay, and change in behavior (increased aggressiveness). Those side effects are mainly due to adverse vaccination administration practices, which are mentioned in another study (29).

Besides vaccination coverage, vaccination effectiveness also remains an important challenge in the Vietnamese context. A study in Tay Ninh province showed that despite a vaccination uptake of 85.4%, the sero-conversion in this province was only 60.6% (30). The imperfect application, storage, and delivery of vaccines can explain the relatively low effectiveness of vaccination (31). Past experiences of vaccine failure can discourage farmers from using it. Advantages of vaccination such as avoidance of animal slaughter, avoidance of carcass disposal, and decreased level of viral excretion (32) are highly relevant to developing countries. However, implementation issues linked to the man-power requirements for post vaccination surveillance and the need for multiple (cumulative) vaccine injections to achieve prophylactic protection (32) can also impair its effectiveness in the field.

Farmers’ perception of the effectiveness of vaccination strongly affects their willingness to implement it (29). Education campaigns that aim at maintaining or enhance farmers’ awareness of the benefits of FMD vaccination should be organized by veterinary authorities before each vaccination campaign (before April or September each year). While some costs related to the awareness campaigns are covered by authorities, like document preparation, invitation letter, speaker invitation, and television program, other costs such as document purchase and time spent in attending trainings, are supported by the farmers. It is estimated that in 1 year, 20 kVND in document and 115 kVND in labor time need to be spent by farmers for participating in education campaigns. Those costs increase the additional costs component and subsequently decrease the BCR of vaccination. Those additional costs might have more effect on the BCR of beef cattle than on the BCR of dairy cattle. Subsidies should be provided by the government to promote farmer’s participation in trainings campaigns (technical document purchase and opportunity cost for attendance) in the form of subsidies should be added in this case. Vaccination in beef cattle could not be disregarded especially in a context of FMD eradication objective.

As specified in our assumptions, our study did not consider the specific impact of chronic FMD. Chronic FMD was reported to reduce milk production by 80% in affected cows (3, 33) and caused some clinical signs such as heat intolerance, infertility and, in general, poor productivity (34). Moreover, the chronic form of FMD usually starts around 4 weeks after the occurrence of the acute form (34), which makes its impact difficult to quantify as Vietnamese smallholder farmers usually do not systematically record cow performance. Quantifying losses due to chronic FMD would require long-term farm surveys. Further studies focusing on the economic impact of FMD at the local-level should consider the chronic form of FMD. A BCA study conducted in Sudan showed that chronic FMD is responsible for 28.2% of the total farm losses due to FMD (3). Therefore, including the impact of chronic FMD would probably increase the estimated saved costs and BCR of FMD vaccination.

It was assumed that cattle infected once by FMD did not get infected later in their productive life. Actually, cattle can be infected in several occasions by viruses of different serotypes (35). The predicted FMD incidences values are, therefore, probably underestimated. Correcting this bias would increase the BCR of FMD vaccination.

The government incentives for vaccination (subsidies) were not taken into account in this analysis in order to simplify the formula and make it conservative. Excluding such subsidies in our analysis enabled us to show that even if vaccination costs are fully supported by farmers, it still generates a positive net return. Currently, only vaccine purchases by small-scale farms are covered 100% by the subsidies whereas larger scale farms already support part of their vaccination costs (subsidies cover vaccine cost for up to 20 cattle). Dairy cattle farms get a higher BCR from FMD vaccination compared with beef farms as losses caused by FMD are higher in dairy farms than in beef farms (36) in the “*status quo*” scenario (without vaccination). Indeed, dairy cows have a higher replacement cost than beef cows, since they are more valuable in terms of performance and productivity.

The cost of the cattle movement restriction, which includes the additional feed intake of unsold animals during the restriction time, was not included in the analysis. According to the Vietnamese government regulation, movement restriction is implemented by the local veterinary authorities upon detection of the first FMD case in the area and is maintained all along the outbreak period. The ban ends 21 days after detection of the last FMD case (9). However, the application of this control measure at the local level might vary from one location to another and accurate data on the implementation of movement restrictions (or delay in selling time for affected farm) are difficult to collect in practice. The inclusion of such parameter would have increased the BCR of FMD vaccination.

The average cattle morbidity rate at the farm-level was around 60%, which is consistent with the results of a case study conducted in Ethiopia (37) but different to the results of another study which found morbidity rates reaching up to 100% (38). In our study, FMD cases were defined by the presence of clinical signs as recorded by farmers. Cattle present in infected farms that did not develop clinical signs were considered healthy. In reality, unapparent infections may occur in cattle whose susceptibility has been reduced by vaccination (38). Moreover, immunized animals subsequently exposed to FMD infection may become chronic carriers without developing clinical symptoms of the disease (16, 39, 40). On the other hand, endemic strains of FMD virus (e.g., serotype O in Vietnam) might cause mild forms of the disease in indigenous Zebu cattle in Asian endemic countries (38). Those aspects could lead to misdiagnosis by farmers and to an underestimation of the mean herd morbidity rate.

The mean FMD mortality in adult cattle observed in our study (12%) was considered higher than the one reported in the literature (7.3%) (2). As a consequence, the mortality variable in literature was used in our calculation instead of the one found by the survey. The possible explanation for the difference between literature data and the survey findings was described as follow. FMD infected animals may have secondary infections during recovery time (digestive troubles, hemorrhagic septicemia, etc.), which could delay or impede their recovery or even lead to their death in some instances. In case cattle do not recover well or die from a secondary infection, they are sent to slaughterhouse, as a consequence of FMD infection, even if FMDV does not directly cause their death. Subsequently, they were reported as death due to FMD to the research team. Moreover, high mortality was mainly observed in dairy farms using highly efficient cattle breeds which are more sensitive to the disease, in comparison to local breeds or crossbreeds used in beef farms. The both value of mortality rate (literature and survey finding) were used as part of the sensitivity analysis and lowering mortality could overestimate the BCR in dairy cattle.

## Conclusion

Our study demonstrated that FMD biannual vaccination strategy is financially and clearly profitable for dairy cattle farmers in Vietnam even if all the vaccination costs are supported by the farmers but not in beef farm. It also showed that FMD vaccination is more profitable for dairy farmers than beef farmers. The results of this study could be used to refine the FMD control program and motivate farmers to use FMD vaccination. A similar study could also be implemented at the national-level to evaluate the BCR of the FMD vaccination strategy and adapt it to achieve the FMD eradication objective in Vietnam. This study’s research framework and results are expected to become a firm ground for further research and awareness program.

## Author Contributions

DT, FG, AD, and MP designed the study, contributed to the analyses, and drafted the manuscript. DT and MP designed the data collection instrument and drafted the manuscript. VG and SB reviewed the results and drafted the manuscript. The manuscript has been read and approved by all authors.

## Conflict of Interest Statement

The authors declare that the research was conducted in the absence of any commercial or financial relationships that could be construed as a potential conflict of interest.

## Abbreviations

NPV, net present value; BCA, Benefit-cost analysis; BCR, benefit–cost ratio; FMD, foot-and-mouth disease; kVND, thousands of Vietnam Dong (Vietnamese currency); NA, not applicable.
