# Supplementary material for: Benefit–Cost Analysis of Foot-and-Mouth Disease Vaccination at the Farm-Level in South Vietnam
Source: Front Vet Sci. 2018 Feb 26;5:26. doi: 10.3389/fvets.2018.00026 (PMC5834816; doi:10.3389/fvets.2018.00026)
Supplement: Supplementary file 1 [file table_1.docx]

**Table 8.** Benefit-cost ratio and sensibility analysis results of BCR for vaccination strategy against foot-and-mouth disease in three production types.

| Scenario | Benefit-cost ratio | | |
| --- | --- | --- | --- |
|  | Small-scale dairy cattle farms | Large-scale dairy cattle farms | Beef cattle farms |
| Baseline model^a^ | 5.24 (1.88-11.61) | 12.34 (2.84-5.75) | 4.91 (0.31-1.95) |
| Vaccination cost ↑25% (C1) | 4.83 (1.83-10.2) | 5.26 (2.73-10.55) | 1.78 (0.72-5.98) |
| Vaccination cost ↑50% (C2) | 4.5 (1.78-9.11) | 4.87 (2.63-9.23) | 1.63 (0.3-3.84) |
| Market price of cattle and milk ↓ 10% (C3) | 4.76 (1.72-10.54) | 5.22 (2.58-11.21) | 1.77 (0.28-4.46) |
| Market price of cattle and milk ↓ 20% (C4) | 4.28 (1.55-9.47) | 4.69 (2.31-10.09) | 1.59 (0.26-4.0) |
| Vaccination cost ↑25% + Market price of cattle and milk ↓ 10% (C5) | 4.39 (1.67-9.27) | 4.79 (2.47-9.60) | 1.61 (0.28-3.92) |
| Vaccination cost ↑50% + Market price of cattle and milk ↓ 10% (C6) | 4.08 (1.63-8.27) | 4.42 (2.39-8.39) | 1.49 (0.27-3.49) |
| Vaccination cost ↑25% + Market price of cattle and milk ↓ 20% (C7) | 3.95 (1.51-8.33) | 4.3 (2.23-8.63) | 1.45 (0.25-3.52) |
| Vaccination cost ↑50% + Market price of cattle and milk ↓ 20% (C8) | 3.67 (1.47-7.43) | 3.97 (2.14-7.55) | 1.33 (0.25-3.14) |

^*^Distribution of the results: mean (CI 95%); ^a^: data from Table 7
